# “Reading A Book, By A Gain In Your Wit”--How to cultivate high school students' sustainable reading: A multi-factor analysis

**DOI:** 10.1016/j.heliyon.2023.e23333

**Published:** 2023-12-03

**Authors:** Rui Wu, Fei Liu, Xiangfu Situ, Wei Huang

**Affiliations:** a School of Education Science, Nanjing Normal University, Nanjing, China; b Nanjing Ninghai High School, Nanjing, China; c School of Teacher Education, Nanjing Normal University, Nanjing, China

**Keywords:** High school students, Reading motivation, Reading self-efficacy, Social support, Reading satisfaction

## Abstract

Reading cultivation is an important task of education. In order to ensure the sustainable reading behavior of high school students, more and more scholars begin to pay attention to the research of reading psychology. In order to explore the psychological mechanism of high school students' reading, based on the theory of self-efficacy and achievement motivation, this study explores the chain intermediary model of 600 high school students' reading motivation influencing reading satisfaction through reading self-efficacy and social support. The results show that there are significant positive predictors among reading motivation, reading self-efficacy, social support and reading satisfaction. At the same time, reading self-efficacy and social support play an intermediary role between reading motivation and reading satisfaction of high school students respectively, and jointly play a chain intermediary role. The research results are of great theoretical and practical significance for understanding the reading psychology of senior high school students and ensuring their sustainable reading behavior.

## 1 Introduction

With the creation of writing, reading emerged accordingly, and it is an integral part of human civilization [1]. Reading is very important in people's usual information exchange. Some scholars believe that reading is the ability of individuals to understand the meaning of written symbols, while others believe that reading should include three levels: sensory, rational and experiential [2,3]. Reading is a unique cultural communication behavior of people [4]. With the advent of the Internet era, the reading environment of human society has changed, reading forms have become more diverse, and people's reading styles have become more diversified [5]. Computers, tablets, and cell phones have become learning tools in the study and life of young people. This has played a big impact on traditional reading [6]. Due to the emergence of the Internet, reading content has also become diverse, leading to many related problems gradually coming to the fore [7]. In particular, research on reading for adolescents has received a great deal of attention worldwide, and reading is seen as one of the most important initiatives to improve student literacy and is a focus of research in the field of education [8]. Journalism, school literature, literary masterpieces, humorous sketches, martial arts novels, and news are all categories of reading [9]. Today's reading texts are becoming more and more popular, reading accompanied by pictures is on the rise, traditional ways of reading are no longer the only way, and online reading is more likely to be sought after by the younger generation [10]. The related research on reading followed.

To meet the reading needs of adolescents and to help them be able to sustain their reading habits, researchers have addressed the psychological and educational aspects of reading for adolescents. Traditionally, adolescents read and write in the sense of applying and understanding words, which is reflected in the four skills of listening, speaking, reading, and writing [11]. With the spread of Internet reading, adolescents need not only the ability to listen, speak, read, and write “text" but also the ability to read and write “Internet" [12]. Research data shows that the United States administered the Comprehensive Assessment Design, a high-stakes test for elementary and middle schools in the 1990s and included online reading skills as an indicator of student learning [13]. Some countries have also included online reading proficiency in their primary and secondary school curriculum standards. Ireland, on the other hand, has introduced new reading competencies for university students, mainly in online reading, problem solving, communication and writing [14]. All these countries have put in place a number of educational policies to ensure that students can maintain their reading habits as long as possible without losing interest in reading due to various factors. This undoubtedly confirms that many countries are paying more and more attention to students' continuous reading. Although there are more and more studies on continuous reading, and many countries have introduced many measures to ensure the possibility of reading, we have to say that there is still little research on students' reading psychology. It is very helpful to explore students' reading psychological mechanism and its improvement methods from this aspect.

As a group of adolescents, the psychological state of reading among high school students has received even more attention. Due to their developmental characteristics, high school students are curious and exploratory in all matters of the outside world. But because of this, they do not yet have adult judgment and need more outside support to help them read satisfactorily, rather than being frustrated and interrupted in their search for reading material [15]. In response, the Chinese government has proposed a number of initiatives related to the language subject, such as increased guidance on reading instruction and more ways for students to read. In addition, scholars from China also believe that the support from schools and teachers is of great significance to students' sustainable development [16,17].

Therefore, in order to investigate the psychological feelings of high school students in the reading process and to ensure better reading satisfaction, this study will explore the mechanism of external support and their own feelings (efficacy) on reading satisfaction in the reading process from the perspective of high school students' own reading motivation. Through this mechanism, it will provide a reference and realistic basis for schools, teachers, and relevant departments to guide students in reading and propose policies related to reading.

## 2 Literature review and research hypothesis

Self-efficacy and achievement motivation theories are common theories for studying motivation, and the theoretical foundation of this study will be based on them. Self-efficacy refers to an individual's presumptions and judgments about his or her ability to perform a behavior [18]. Bandura defines self-efficacy as the degree to which people feel confident in their ability to use the skills they possess to perform a given work behavior [19]. Bandura believes that in addition to outcome expectations, there is an expectation of efficacy. Outcome expectancy refers to a person's presumption that a certain behavior will lead to a certain outcome [20]. If a person predicts that a particular behavior will lead to a particular outcome, then that behavior may be activated and selected. Bandura argues that because of the variability between different areas of activity, the competencies and skills required vary widely [18]. A person's self-efficacy is different in different areas. Therefore, there is no general sense of self-efficacy. Whenever self-efficacy is discussed, it refers to the self-efficacy associated with a particular domain. Obviously, in the reading process, reading self-efficacy cannot be ignored, but is one of the most important factors to be studied [21].

Achievement motivation is an individual's quest to maximize individual value or to achieve the most perfect state through methods in the pursuit of self-worth [22]. It is a manifestation of an intrinsic drive, but also has the ability to directly influence a person's behavioral activities, the way of thinking, and is a long-term state [23]. David C. McClelland, a professor at Harvard University, proposed the theory of achievement motivation in a series of articles in the 1950s through his research on human needs and motivation [24]. McClelland categorized people's higher-level needs as the need for achievement, power, and affinity [25]. He did an in-depth study of these three needs, especially the need for achievement [26]. John William Atkinson, an American psychologist, further deepened McClelland's achievement motivation theory in 1963 and proposed the widely influential achievement motivation model [27]. Achievement motivation is the motivation of people who want to engage in activities that are important to them, difficult and challenging, in which they can achieve perfect and excellent results and accomplishments and can surpass others [28]. As a prerequisite for sustained reading, reading motivation must be one of the prerequisites for subsequent sustainable reading behaviors, and therefore, reading motivation will also be the independent variable in this study for subsequent research.

Some scholars who have studied achievement motivation theory have identified self-efficacy as a positive component of achievement motivation and have even proposed replacing achievement motivation with self-efficacy as an explanatory factor for human behavior [29]. Other scholars have argued that self-efficacy and achievement motivation together serve as prerequisites for achievement goals, indirectly influencing achievement behavior [30]. For example, Elliot in his study classified achievement goals into three orientations: mastery goal, performance approach, and performance avoidance goal [31]. It was found that there was a significant positive relationship between self-efficacy and the first two goals and a significant negative relationship between performance avoidance goals [32]. Thus, in this study, when studying high school students' psychological states of reading, motivation to read was used as the independent variable and satisfaction with reading as the dependent variable, while the mechanisms of self-efficacy and other external factors operating were considered. In this way, it is possible to adequately discuss whether high school students will choose to converge or avoid reading in the future.

### 2.1 Reading motivation and related studies

So far, scholars have not provided a clear definition of reading motivation, and different scholars have provided different explanations for reading motivation depending on their research perspectives and focus [33]. According to some scholars, reading motivation refers to an individual's goals and beliefs about reading [34]. While Kristin Conradi et al. point out that reading motivation is a combination of internal drivers of reading goals and individual beliefs and attitudes [35]. Wieldand Cambria pointed out that the expected value theory of reading motivation encompasses both self-control and self-achievement values of students [36]. Christopher A. Wolters et al. argued from the viewpoint of achievement goal theory that reading motivation can include reading mastery goal, reading task completion goal reading task avoidance goal [37]. Analysis of existing research on the influence of reading motivation reveals that it can be broadly divided into two categories: individual differences influence and environmental influence.

Regarding individual differences. First, researchers differ in terms of gender differences. Some studies have shown that, in general, girls are more motivated to read than boys, value reading more, have a stronger sense of belief, and have significant gender differences in reading motivation. Others have argued that there are no differences in reading motivation by gender [38]. On the contrary, the results of one study found that boys and girls showed the same level of motivation on school reading activities related to reading ability and reading value, with no gender differences. Second, in terms of grade differences. The results of a study from Hong Kong found significant grade differences in all dimensions of reading motivation [39]. In addition, some researchers have revealed that there are differences in reading motivation in terms of age. For example, Unrau et al. found through a survey study that both internal and external motivation to read gradually diminished with age, only that internal motivation diminished to a greater extent than external motivation [40]. Third, regarding the psychological characteristics of the individual, some researchers have suggested that a child's personality traits have a greater impact on his internal motivation to read [41]. Jan Retelsdorf et al. concluded through their study that reading self-concept can have a greater impact on reading motivation and that there is an interaction between reading achievement and reading self-concept [42]. Natalie Förster et al. found that reading motivation is influenced by personal hope psychology and that human heart hope psychology has an important role in reading achievement [43].

Regarding environmental influences. Since reading motivation has been studied mainly in the adolescent population, environmental influences include mainly the school environment and the home environment. With regard to school environment factors, researchers have found through their studies that teacher support and peer involvement have a positive impact on the development of students' independent reading motivation [44,45]. In terms of home environment factors, researchers have found that family cultural capital has a direct and important influence on students' motivation to read and contributes to the development of students' reading interests [46], and that students with high family economic status are significantly more motivated to read than students with low family economic status [47]. Other researchers have pointed out that parental support styles have an important influence on adolescents' motivation to read and that parental expectations and emotional support play an important role in the development of children's motivation to read [48,49]. Thus, it can be seen that the family environment is another important factor that influences individual motivation to read and has a greater impact on motivating and enhancing children's and adolescents' motivation to read.

### 2.2 Reading self-efficacy and its related research

Reading self-efficacy builds on self-efficacy and academic self-efficacy, and research on self-efficacy and academic self-efficacy has directly contributed to theoretical research on reading self-efficacy. Reading self-efficacy is an expression of academic self-efficacy throughout students' reading activities [21]. The definition of reading self-efficacy can be broadly divided into three perspectives, depending on the focus of the researcher. First, reading self-efficacy is an individual's judgment of his or her ability to complete a reading task; second, reading self-efficacy is an individual's confident evaluation of his or her ability to use reading skills effectively; and third, combining the first two views, reading self-efficacy consists of two components: an individual's subjective judgment of his or her ability to successfully complete a reading task and his or her level of confidence in using reading skills [50].

The American psychologist Bandura's concept of self-efficacy has been validated with support from different disciplines as soon as it was proposed [51]. The study of reading self-efficacy when self-efficacy is applied to the field of education is an important research direction. Many researchers in Western psychology have recognized the existence of this complex relationship through empirical research examinations. Reading is not only a cognitive activity, but also an emotional activity, it is an emotional communication and interaction between the individual reading and the text read, and the emotional component of the individual affects every stage of the reading process [52]. In the reading process, the student's attitude becomes particularly important. If a student is confident in his or her reading ability level, he or she will consider reading to be an easy task; however, if the student is always skeptical about his or her reading ability, feels that he or she cannot read and cannot understand the meaning of the text he or she is reading, then over time, he or she will become fearful and bored with reading [53]. Thus, students who believe in their reading ability have higher reading scores than those who doubt their reading ability, and they are more likely to enjoy the reading process and successfully complete the reading task [54]. Self-efficacy research can be applied to the reform of reading instruction, which will be a revolution in reading instruction [55].

In Chinese reading education, the beginning stage is in elementary school, the developmental stage is in middle school, and the maturity stage is in high school, so high school students should already have good reading skills. But the fact is not what we imagine, in the actual reading teaching process, although some of the high school students already have better reading ability, but there are still a large proportion of students with average reading performance, reading self-efficacy is also not strong, and even many students' reading performance is not satisfactory, they feel bored and afraid of reading. Therefore, choosing the measurement of high school students' reading self-efficacy as a starting point can provide a good understanding of the current situation and development pattern of high school students' reading self-efficacy, identify the causes of low reading self-efficacy, and improve students' reading self-efficacy through a series of effective means to help students improve their reading comprehension and reading achievement, which will play a positive role in high school students' academic as well as personality development.

### 2.3 Reading satisfaction and its related studies

Reading satisfaction, which has been proposed by scholars in recent years, refers to a psychological state that is the result of values, attitudes, emotions, and interactions with the outside world during an individual's reading process [56]. Its main research focuses on three aspects: engagement, supportive relationships, and reading interaction.

First, about the degree of participation. The relationship between students' engagement and learning satisfaction was studied by Elbro et al. who concluded that the degree of student engagement was related to students' participation expectations, effort, interpersonal relationships, and effective reading interactions. level, interpersonal relationships, and effective learning practices were positively correlated; therefore, positive teacher-student relationships, quality support services, and good teaching practices promote student satisfaction while enhancing student engagement [57].

Second, about supportive relationships. The supportive relationship is one of the important factors that cannot be ignored in the learning process. Taking high school students as an example, they are mainly supported from school and home in the learning process. It has been confirmed that when students perceive better school support and parental support, they are able to perform better in their academic performance. Therefore, when students receive better support, they tend to demonstrate a more positive state of learning, and in this way also promote better satisfaction. This is clearly true for the reading process as well.

Third, on reading interactions. Human interaction will be the transmission of influence between people, who are influenced by others while also influencing others in turn [58]. Numerous studies in the field of sociology have proven that in social activities, individual members respond to interactive stimuli with others, changing their personal behavior and perspectives, and consequently having an impact on the performance of the activity [59]. Therefore, reading as a micro-social activity, the reading cognition, thinking, emotion, and decision making of its participating users will also necessarily change as they interact with others. To explore the internal connection between the complex reading interaction behavior of users and the characteristics of users' psychological activities, and to reveal the influence law of reading interaction on users' reading experience from the surface to the inside, it will be beneficial for promoters to make scientific analysis and prediction on the state of promotion environment in the process of reading promotion, and then to formulate practical promotion principles and strategies to achieve promotion goals efficiently, so as to enhance users' reading satisfaction and improve promotion quality and service effectiveness. It will provide a scientific and reasonable basis for enhancing users' reading satisfaction and improving service quality and efficiency.

### 2.4 Research hypothesis

From the above findings, it is clear that the results of these studies reflect a possible stronger relationship between reading motivation, reading self-efficacy, and reading satisfaction. Also, the existing studies on all three variables mentioned that social support (school support, parental support) played a positive influence on the relevant variables. Thus, the study of antecedents of reading satisfaction, guided by self-efficacy and achievement motivation theories, is also well justified. Moreover, these findings also provide a more favorable frame of reference for the four variables of this study.

**Fig. 1:**
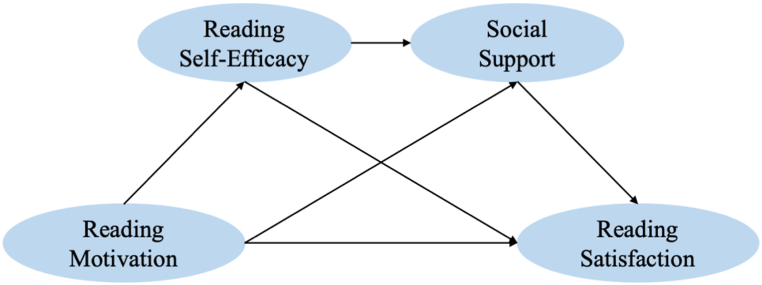
Research hypothesis model.

Therefore, in summary, the following hypotheses are made in this study and the research hypothesis model can be seen in Fig. 1.

● Research Hypothesis 1: There is a two-by-two significant correlation between reading motivation, reading self-efficacy, social support, and reading satisfaction.
● Research hypothesis 2: Reading self-efficacy plays a mediating role between reading motivation and reading satisfaction.
● Research hypothesis 3: Social support mediates the relationship between reading motivation and reading satisfaction.
● Research hypothesis 4: Reading self-efficacy and social support play a chain mediating role in the relationship between reading motivation and reading satisfaction.

## 3 Methodology

### 3.1 Participants

To investigate the mechanism of the role of sustainable reading in high school students, this study conducted a cross-sectional survey of 600 high school students from Jiangsu Province, China, from December 2022 to March 2023. All participants will read about the purpose of collection, intention of use, and informed consent before completing the questionnaire, and by completing the questionnaire, they agree to the use of the data. The study promised that all information would be kept strictly confidential and used for scientific research only. A total of 600 data submissions were finally collected for this study, and after data screening a total of 548 valid data were included in the analysis, with an effective rate of 91.33 %. Among them were 175 male and 373 female students. Due to the restricted distribution time of the sample, this sample was all from the senior class.

### 3.2 Research tools

#### 3.2.1 Reading motivation scale

Reading motivation scale consists of 27 items, which are reading interest (6 items, α = 0.921), social interaction (5 items, α = 0.930), emotional expression (5 items, α = 0.935), information acquisition (5 items, α = 0.916), and personal cultivation (6 items, α = 0.941), with five dimensions [60]. The scores ranged from “not at all" to “very much", all of which were positive, with higher scores indicating higher reading motivation and vice versa. After the questionnaires were collected, the reliability of the reading motivation scale was tested, and the results showed that the alpha coefficient of Cronbach in this study was 0.977. The model fit indices were χ^2^/df = 5.528, RMSEA = 0.091 GFI = 0.800, CFI = 0.917, and TLI = 0.903, which indicated that the scale had high reliability and good stability and consistency. which indicates that the scale has high reliability and good stability and consistency.

#### 3.2.2 Reading self-efficacy scale

Reading self-efficacy was measured using the Reading Self-Efficacy Questionnaire for High School Students, which mainly consists of 29 items, namely, sense of competence (6 items, α = 0.943), sense of control (5 items, α = 0.938), sense of benefit (6 items, α = 0.949), self-confidence (6 items, α = 0.947), planning (4 items, α = 0.926), and resistance to interference (2 items, α = 0.850), a total of six dimensions (of which the 16th item is a lie detector question), using the Likert A five-point scale was used [61]. The scores ranged from “not at all" to “very much", all of which were positive, and higher scores indicated higher reading self-efficacy, and vice versa. After the questionnaires were collected, the reliability of the reading self-efficacy scale was tested, and the results showed that the alpha coefficient of Cronbach in this study was 0.987. The model fit indices were χ^2^/df = 5.908, RMSEA = 0.095, GFI = 0.770, CFI = 0.920, and TLI = 0.908, which indicated that the scale had high reliability and good stability and consistency. indicating that the scale has high reliability and good stability and consistency.

#### 3.2.3 Reading satisfaction scale

Reading satisfaction scale was used in this study, which consists of five questions and only one dimension, and uses a five-point Likert scale. From “not at all" to “very much", all scores are positive, and the higher the score, the higher the reading satisfaction, and vice versa. After the questionnaires were collected, the reliability of the reading satisfaction scale was tested, and the results showed that the alpha coefficient of Cronbach in this study was 0.918. The model fit indices were χ^2^/df = 5.388, RMSEA = 0.090, GFI = 0.984, CFI = 0.991, and TLI = 0.978, which indicated that the scale had high reliability and good stability and consistency. indicating that the scale has high reliability and good stability and consistency.

#### 3.2.4 social support scale

The social support scale incorporated two main dimensions, school support and parental support, according to the needs of this study, with a total of 18 items, including: school support scale (8 items, α = 0.984) and parental support scale (10 items, α = 0.962), using a five-point Likert scale [62]. The scale was scored positively from “not at all" to “very much", with higher scores indicating higher social support, and vice versa. After the questionnaires were returned, the reliability of the social support scale was examined, and the results showed that the alpha coefficient of Cronbach was 0.971. The model fit indices were χ^2^/df = 5.357, RMSEA = 0.089, GFI = 0.893, CFI = 0.966, and TLI = 0.956, which indicated that the scale had high reliability and good stability and consistency.

### 3.3 Analysis method

To test the research hypotheses of this study, the following process was conducted. First, the validity of reading motivation, reading self-efficacy, social support, and reading satisfaction were assessed by using reliability tests and validated factor analysis in SPSS 25.0 and Amos 26.0. Second, all topics were included in exploratory factor analyses to test for common method bias. Third, research hypotheses 1, 2, 3, and 4 were tested by multiple linear regression analysis. fourth, the hypothesized models were tested for validity by Amos 26.0. In addition, the hypothesized model was tested for the significance of mediating effects by Bootstrap method (5000 replicate draws).

## 4 Results

### 4.1 Common method deviation test

Since the data for this survey were obtained from the self-reports of high school students, common methodological biases are possible and need to be tested before continuing with the later analysis. Therefore, the Harman one-factor test was used to test for bias in the variables. The results showed that the eigenvalues of the eight factors were greater than one and the explanatory power of the first factor was less than 40 % of the critical value (variance value of 33.08 %). Thus, common methodological biases do not affect the data results.

### 4.2 Descriptive statistics and correlation analysis

According to the analysis results in Table 1, the mean score of reading motivation was 3.580 with a standard deviation of 1.057. The mean score of reading self-efficacy was 3.669 with a standard deviation of 1.050. The mean score of social support was 3.501 with a standard deviation of 1.169. The mean score of reading satisfaction was 3.594 with a standard deviation of 1.092. the results of correlation analysis showed that reading motivation of high school students was significantly positively correlated with reading self-efficacy, social support, and reading satisfaction (p < 0.001). Reading self-efficacy of high school students was significantly positively correlated with social support and reading satisfaction (p < 0.001). Social support of high school students was significantly positively correlated with reading satisfaction (p < 0.001).

**Table 1:** Descriptive statistics and correlation analysis.

	Mean	Standard Deviation	1	2	3	4
1. Reading motivation	3.580	1.057	1			
2. Reading self-efficacy	3.669	1.050	0.732**	1		
3. Social support	3.501	1.169	0.545**	0.580**	1	
4. Reading satisfaction	3.594	1.092	0.670**	0.818**	0.723**	1

Note: **: p < 0.001.

### 4.3 Mediating effects

Hayes and Scharkow (2013) stated that attention should be paid to the credibility of indirect effects in statistical mediation analyses, and they recommended bias-corrected bootstrap confidence intervals as the most trustworthy test [63]. Therefore, in this study, reading motivation was used as the independent variable, reading satisfaction as the dependent variable, and reading self-efficacy and social support as mediating variables; stepwise regression was used to analyze the mediating effects of reading self-efficacy and social support. The results are shown in Table 2.

**Table 2:** Descriptive statistics and correlation analysis.

No.	Dependent variable	predictive variable	*R*^2^	*β*	*T*	*F*
regression equation (1)	Reading satisfaction	Reading motivation	0.448	0.670	21.096 ***	445.045 ***
regression equation (2)	Reading satisfaction	Reading motivation	0.679	0.154	3.296***	578.763 ***
		Reading self-efficacy		0.705		
regression equation (3)	Social support	Reading motivation	0.366	0.260	5.775***	158.685***
		Reading self-efficacy		0.390		
regression equation (4)	Reading satisfaction	Reading motivation	0.762	0.059	0.388 ***	585.422***
		Reading self-efficacy		0.563		
		Social support		0.364		

As can be seen in Table 2, the results of equation (1) indicate that reading motivation significantly and positively predicts reading satisfaction (p < 0.001). The results of equation (2) indicated that both reading motivation and reading self-efficacy significantly and positively predicted reading satisfaction (p < 0.001). The results of equation (3) indicated that both reading motivation and reading self-efficacy significantly and positively predicted social support (p < 0.001), thus testing hypothesis 2. The results of equation (4) indicated that both reading self-efficacy and social support also played a significant role when included in the relationship between reading motivation and reading satisfaction (p < 0.001), thus testing hypotheses 3 and 4. Meanwhile, the R2 value of the independent variable reading motivation on the dependent variable reading satisfaction increased from 44.8 % to 76.2 %, the R2 changed to 31.4 %, and the regression coefficient of the independent variable on the dependent variable decreased from 0.670 to 0.059; moreover, the regression coefficient was also significant. Thus, high school students' motivation to read can indirectly influence reading satisfaction through the mediating effects of reading self-efficacy and social support.

Following immediately, this study further tested the significance of the mediating effect using the bias-corrected Bootstrap method (5000 repetitions) and found that reading self-efficacy did mediate between reading motivation and reading satisfaction, with a standardized effect value of 0.426 for path 1, and a 95 % confidence interval (0.349, 0.510), which did not included 0, demonstrating a significant mediating effect and a relative mediating effect of 61.47 %. Social support mediated the relationship between reading motivation and reading satisfaction with a standardized effect value of 0.098, 95 % confidence interval (0.051, 0.153) for path 2, which did not include 0, demonstrating a significant mediating effect and a relative mediating effect of 14.14 %. The standardized effect value for the chain mediating effect of reading self-efficacy, social support between reading motivation and reading satisfaction was 0.107, 95 % confidence interval (0.069, 0.152), excluding 0. The chain mediating effect was significant, and the relative mediating effect was 15.44 %. The specific results are shown in Table 3.

**Table 3:** Results of intermediary path coefficient analysis.

	Effect	Boot Standard Error	95 % Confidence Interval		p	Relative Mediating Effect
			Boot LLCI	Boot ULCI		
**Total Effect**	0.693	0.033	0.628	0.757	0.000	/
**Direct Effect**	0.062	0.032	0.002	0.125	0.009	/
**Total Mediating Effect**	0.631	0.042	0.552	0.716	0.000	91.05 %
**Path 1**	0.426	0.041	0.349	0.510	0.000	61.47 %
**Path 2**	0.098	0.026	0.051	0.153	0.000	14.14 %
**Path 3**	0.107	0.021	0.069	0.152	0.000	15.44 %

Note: Path 1：Reading motivation-Reading self-efficacy-Reading satisfaction; Path 2：Reading motivation-Social support-Reading satisfaction; Path 3：Reading motivation-Reading self-efficacy-Social support-Reading satisfaction.

**Fig. 2:**
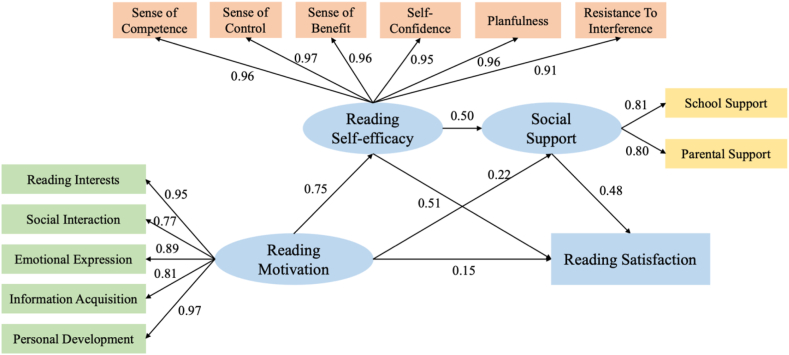
Intermediary model.

Finally, this study imported the data into Amos for structural equation modeling and produced the results shown in Fig. 2 below. It was found that reading self-efficacy and social support can play a chain mediating role between reading motivation and reading satisfaction, and the above findings were further validated. Furthermore, this study compared the hypothetical model data and found that χ2/df = 5.213, RMSEA (root mean square error of approximation) = 0.088, NFI (standard fit index) = 0.966, IFI (incremental fit index) = 0.972, CFI (comparative fit index) = 0.972, GFI (goodness-of-fit index) = 0.912, and AGFI (adjusted GFI) = 0.866, all of which meet the requirements and indicate that the model fits well.

As seen in Fig. 2, the mediating effect of high school students' reading self-efficacy and social support between reading motivation and reading satisfaction was 0.631, the direct effect of reading motivation on reading satisfaction was 0.062, and the total effect was 0.631 + 0.061 = 0.693. The proportion of the mediating effect to the total effect was 91.05 %.

## 5 Discussion

### 5.1 Analysis of high school students' reading situation

Through the survey and statistics on the reading situation of high school students from China, we found that the most important way of reading for high school students is through cell phones and Kindle and paper books; the most read content is youth literature novels, followed by geography and military magazines; the preferred reading place is home; the main way to get reading materials and resources makes to go to bookstores to buy them, but downloading through the Internet or However, downloading or purchasing reading resources through the Internet is also an important way for them to obtain reading materials.

From the previous data analysis, it is known that there is no significant difference in reading motivation, reading self-efficacy, social support, and reading satisfaction by gender, which means that high school boys and girls have equal levels of online reading motivation and reading self-efficacy. In terms of parents' education level, there are significant differences in reading motivation, reading self-efficacy, social support, and reading satisfaction, and the family is the child's first school. The parenting style in the family of origin affects the child's learning, personality, and all aspects of life. Parents who are academically successful give their children confidence in their studies, and this is where vicarious experiences come into play. Parents with higher education also influence the way they teach their children. Parents with higher education tend to be more involved in their children's learning, pay attention to their children's learning, give encouragement to their children, and discuss knowledge with their children, while parents with lower education tend to be less involved in their children's learning and may not know which is the better way to teach their children compared to parents with higher education, and They may not be able to set a good example for their children, and therefore give little guidance to their children in education. It can be concluded that parents' educational level determines their effectiveness in educating their children, which in turn affects their children's academic self-efficacy. Therefore, the conclusion of this study that parents' educational level has a significant effect on their children's reading reminds us once again that we should pay attention to the important influence of parents on their children's education, and that parents should do a better job of setting a good example for their children's learning, taking effective measures to stimulate their children's interest in learning, and participating in their children's learning as much as possible.

### 5.2 Relationship between high school students' reading motivation and reading self-efficacy

The results of the data revealed that reading interest, social interaction, emotional expression, information acquisition, and personal cultivation were significantly and positively related to students' reading self-efficacy. Some studies have confirmed that in addition to traditional paper books, the reading methods chosen by adolescents in the reading process are more often digital resources such as e-books, digital newspapers, databases, literary works, and network news with the Internet as the carrier, and e-books have become one of the main forms of digital reading for adolescents in the information age, and the content of online reading is mainly leisure and entertainment, fiction books, educational knowledge, and other. The content of online reading is mainly based on leisure and entertainment, fiction books, educational knowledge, and other contents. Therefore, e-books, as another form of reading for today's adolescents, not only encompass the advantages of paper-based reading in terms of sensory and book content integrity, but also have the characteristics of portability and more convenience in searching and downloading books. E-books on the one hand is conducive to students to use fragmented time to read, on the other hand can let young you that according to their own interests and preferences to choose reading content, on the other hand can also stimulate young people's interest in reading, accept new ideas, and promote the development of their thinking.

Internet information era, teenagers reading process, online resources through pictures, audio, video, and other ways to attract the attention of young people, vivid and interesting, convenient, and fast e-book fun advantage is obvious. When carrying out reading, adolescents can not only read through the traditional text, graphics, but also listen to audiobooks, use and play audio, video, etc. The reading interface is made exquisite and beautiful, pleasing to the eye, smooth operation, with humane, so many points naturally improve the interest of adolescents in reading. It is said that interest is the best teacher, so through the improvement of reading interest, thus can increase the self-efficacy of youth reading. In recent years, more and more people understand and gradually accept the text format of this kind of graphical information. Through reading, adolescents can also quickly and effectively access the resources they want. Problems that are not understood in time in the classroom can be understood by looking for information online, especially for students who are introverted and are afraid to ask their teachers or classmates about problems they do not understand, so it is a good way to check the Internet after class. By collecting information online, adolescents stimulate their creative thinking and promote their sense of independent learning, which further enhances their reading self-efficacy. In the process of reading, adolescents can communicate with others and form multiple communication groups, and in the communication with others can collide with different sparks, thus exercising their thinking and expanding their interpersonal communication, which makes them love reading more. At the same time, through reading, can also promote the personal cultivation of young people, a good work, can teach people to distinguish between right and wrong, in a low mood or other negative emotions, online reading is good for readers to relax themselves, timely relief of stress, so that they can be in a better state of mind to study and life.

### 5.3 Relationship between reading self-efficacy and reading satisfaction among high school students

The correlation analysis shows that reading self-efficacy is significantly and positively related to reading satisfaction. In the regression analysis we found that the subjects' reading self-efficacy had a significant positive predictive effect on reading satisfaction, with the greatest predictive power for sense of control, sense of benefit, self-confidence, and resistance to interference. This indicates that students with high self-efficacy, sense of benefit, self-confidence, and resistance to interference in their usual reading activities will read at a higher level and have higher self-perceived satisfaction. Thus, in students' usual reading activities, we can promote the improvement of adolescents' reading self-efficacy by taking effective means to stimulate their interest in reading, so that they can feel that they have learned through reading, enhance their understanding of what they have learned and learn to integrate it, learn to make reasonable plans according to their actual situation, and enhance their confidence level in their reading ability. At the same time, they must also develop their ability to focus on learning and improve their resistance to interference. Therefore, in the teaching process, on the one hand, we need to enhance students' reading self-efficacy from different perspectives, fully explore the differences of each student, pay attention to students with poor reading performance, find out their root causes, in order to prescribe the right remedy, stimulate their interest in reading, awaken their will to read, and cultivate good habits that enable them to read independently.

On the other hand, the increase in reading satisfaction will in turn enhance students' self-efficacy, strengthen their confidence in reading, and make them less averse or fearful of reading, so that they are more willing to devote more time to reading than others and strive for success. This shows that the development of reading self-efficacy is an effective way to enhance students' reading quality, improve their reading satisfaction and develop good reading habits.

### 5.4 The chain mediating role of reading self-efficacy and social support in the relationship between reading motivation and reading satisfaction

The results of analyzing the data showed that reading self-efficacy and social support played a significant chain mediating role in the relationship between reading motivation and reading satisfaction, indicating that reading motivation mainly influences students' reading self-efficacy, with the further help of social support, which in turn influences their reading satisfaction. The fact that self-efficacy can influence students' satisfaction is an issue on which different psychologists have different views and their studies have their own focus. (Two papers on how self-efficacy affects student satisfaction are included here). By analogy, then, the same is true for the relationship between reading self-efficacy and reading satisfaction.

After summarizing the previous research results, it can be found that there are still relatively few studies on reading motivation, and they are mainly focused on studies of college students, and the previous studies are more focused on enhancing reading behavior, and there are fewer observations on reading psychology. This study explored some of the mechanisms of reading psychology by exploring the relationship between reading motivation and reading self-efficacy, social support, and reading satisfaction in high school students. Therefore, educators as well as parents can promote students' reading self-efficacy by supporting their reading and guiding them to use various resources to support their reading, which in turn can improve their reading satisfaction.

## 6 Educational practice assumption

Based on the analysis of these results, it is clear that reading self-efficacy is an important factor that schools and teachers can focus on in the educational process. For this reason, the authors of this study tried to proposed a “three-level reading theory(TLRT)" based on their long experience in practice, with the aim of contributing to reading self-efficacy. It is worth mentioning that this theory has been applied and tested in China, and the reason why this study proposes this educational practice assumption is to get more attention from scholars and educational practitioners.

In the process of reading, progressive reading can help students to develop a clear sequence of reading learning around a topic or core concept, so that students can optimize and upgrade their knowledge, ability, character, and literacy. TLRT provides the theoretical basis and practical direction for this. TLRT includes three levels: “interpretation, decoding and evaluation". The interpretation points to reading for understanding, focusing on “how to read for understanding" and “how to read better". The decoding points to reading through, focusing on “how to read well and deeply". The evaluation points to reading through, focusing on “how to read through" and “how to read “I think"", guiding students to read critically and critically to reconstruct meaning. The theory uses a ladder approach to provide an operational teaching procedure for reading instruction, allowing students to master reading strategies and methods, develop reading skills, and achieve literacy progression in the process of learning progression.

Contemporary research in language teaching theory suggests that reading and writing share common cognitive strategies and are two similar, dynamic, and interacting processes, both containing original memory structures, both analyzing discourse structures, and both involving the acts of comprehension and creation. Accordingly, we designed a three-level model of “reading and writing progression" based on the TLRT.

The first level is text interpretation, which points to basic reading and writing. This level guides students to learn to obtain text information, understand text content, grasp the main idea of the text, experience the emotion of the text, and be able to combine what they have learned to accomplish basic writing goals. Accurate use of disciplinary language in single-subject reading, writing, and discussion can help students learn in depth and better construct their own disciplinary understanding. Understanding and mastering the linguistic features and reading strategies of different disciplines and being able to use the expressions of the discipline for writing expressions and communication has a very good effect on facilitating disciplinary learning.

The second level is symbolic processing, which points to fine-grained reading and writing. Symbolic code includes encoding and decoding, decoding points to reading and encoding points to writing. The decoding means aesthetic reading, to understand the techniques and arts in the text, to read the author's unique thinking and the art of expression, and to read the wisdom of speech. Then, coding points to professional writing, where students are prompted to mobilize their accumulated knowledge, skills, and experience through learning tasks, and to construct meaning aesthetically and systematically according to the disciplinary thinking and logic of different disciplines, matching the corresponding disciplinary expressions and structural approaches.

The third level is the aesthetic and discursive reading and writing, which points to creative reading and writing. At this level, students can express their own opinions and views by critiquing, evaluating, and parsing the text, mainly emphasizing readers' critical thinking and creative reconstruction of the text. On the platform of the literacy workshop, students are free to use intertextual comparative reading, group textual reference reading, inquiry and research reading, and multi-perspective conversion reading to discover the deeper value implications of the text. Nowadays, information is presented in a more diverse and complex way, and students need to deal with not only text, but also images, tables, data, audio, video and so on. With the vast amount of information available, students must also learn to discern the authenticity, reliability, and value orientation of information in order to reconstruct their own knowledge systems and values, and to create new knowledge with the help of diverse information texts. The democratic, open, communicative, and collaborative format of the Reading and Writing Workshop is conducive to the development of students' critical thinking, communicative and collaborative skills, and creative abilities.

The three levels of steps point to the goals of reading and writing instruction, i.e., consolidating the foundation, grasping the key, and moving to the higher level, thus achieving the progressive development of literacy. This is also the area that this study hopes to further investigate in the future in the teaching practice of reading based on the analysis of reading psychology.

## 7 Limitations

Although this study has obtained some valuable results, it is still affected by some subjective and objective factors, as follows.

(1) Due to the heavy workload of high school students, the time that can be reserved for the subjects to fill out the questionnaire is only 15 min, which may have some influence on the students' true responses.
(2) Survey data for sophomore and junior students were not available. Because sophomores and juniors are under academic stress and many of them are unable to participate in the questionnaire, only the sample of seniors was included in this study, and the sample data of the other two grades were lacking.
(3) In terms of sample sampling, the high schools investigated in this study are better middle schools, where students generally have higher academic performance and generally better study habits, so the external validity of this study needs to be verified in a larger scale. In future studies, the sampling range can be appropriately expanded to make the sample more representative.
(4) Students' reading satisfaction is obviously also influenced by other factors. This study examined reading satisfaction using a self-administered scale that focused on self, school, and parental factors according to the needs of the study, but students' satisfaction is influenced by other factors such as logical thinking skills, reading habits, and reading volume in addition to reading motivation, reading self-efficacy, and social support, and these additional variables were not validated in this study.

## Ethics statement

The study was conducted in accordance with the Declaration of Helsinki and approved by the Institutional Review Board of Nanjing Ninghai High School (protocol code No.20230505 and January 3, 2023 of approval). At the same time, because the research participants of this study is high school students, and some of the research participants are under 18 years old, this study began after the joint consent of school leaders, teachers and parents.

## Consent statement

Participants’ informed consent has been obtained for this study.

## Data availability statement

Data is available upon reasonable request.

## Additional information

No additional information is available for this paper.

## Funding statement

This research was funded by Major Project of Jiangsu Education Science Planning, grant number A/2021/07; Jiangsu Provincial Education Science Planning Key Topics, grant number D/2021/02/31; Jiangsu Provincial Education Science Planning Research Project, grant number C-6/2020/01/28; Nanjing Education Science Planning Research Project, grant number LZD/2021/101.

## CRediT authorship contribution statement

**Rui Wu:** Writing – review & editing, Writing – original draft, Visualization, Validation, Software, Resources, Methodology, Investigation, Funding acquisition, Formal analysis, Data curation, Conceptualization. **Fei Liu:** Writing – review & editing, Writing – original draft, Software, Methodology, Formal analysis, Data curation. **Xiangfu Situ:** Software, Methodology, Data curation. **Wei Huang:** Writing – review & editing, Writing – original draft, Supervision, Funding acquisition.

## Declaration of competing interest

The authors declare the following financial interests/personal relationships which may be considered as potential competing interestsRui Wu reports article publishing charges was provided by Nanjing Normal University. If there are other authors, they declare that they have no known competing financial interests or personal relationships that could have appeared to influence the work reported in this paper.
